# Analysis of Metabolites in Stem Parasitic Plant Interactions: Interaction of *Cuscuta–Momordica* versus *Cassytha–Ipomoea*

**DOI:** 10.3390/plants5040043

**Published:** 2016-12-07

**Authors:** Takeshi Furuhashi, Takemichi Nakamura, Koji Iwase

**Affiliations:** 1 Department of Natural and Environmental Science, Teikyo University of Science, 2-2-1 Senju-sakuragi, Adachi, Tokyo 120-0045, Japan; kiwase@ntu.ac.jp; 2 Molecular Structure Characterization Unit, RIKEN CSRS, 2-1 Hirosawa, Wako, Saitama 351-0198, Japan; takemi@riken.go.jp

**Keywords:** plant interaction, *Cuscuta*, *Cassytha*, stem parasitic plant

## Abstract

*Cuscuta* and *Cassytha* are two well-known stem parasitic plant genera with reduced leaves and roots, inducing haustoria in their stems. Their similar appearance in the field has been recognized, but few comparative studies on their respective plant interactions are available. To compare their interactions, we conducted a metabolite analysis of both the *Cassytha–Ipomoea* and the *Cuscuta–Momordica* interaction. We investigated the energy charge of the metabolites by UFLC (ultra-high performance liquid chromatography), and conducted GC-MS (gas chromatography-mass spectrometry) analysis for polar metabolites (e.g., saccharides, polyols) and steroids. The energy charge after parasitization changed considerably in *Cassytha* but not in *Cusucta*. *Cuscuta* changed its steroid pattern during the plant interaction, whereas *Cassytha* did not. In the polar metabolite analysis, the laminaribiose increase after parasitization was conspicuous in *Cuscuta*, but not in *Cassytha*. This metabolite profile difference points to different lifestyles and parasitic strategies.

## 1. Introduction

Stem parasitic plants develop haustoria and suck nutrients from host plants. *Cuscuta*, *Cassytha*, and *Viscum* are three commonly known stem parasitic plant genera [1]. Among these plants, *Cuscuta* and *Cassytha* have a very similar appearance and share common characteristics. For example, the haustoria-forming region is located on the stem, and the haustoria are induced by light and contact cues, requiring no chemical cues [2,3]. Moreover, both have highly reduced scale leaves. Nonetheless, there are certainly differences in taxonomy and also lifestyle between these two plants: *Cassytha* belongs to the family Lauraceae and *Cuscuta* to the Convolvulaceae [4,5]. *Cassytha* seedlings develop roots, but *Cuscuta* has only a root vestige for a few days after germination [6]. Consequently, *Cuscuta* as a holoparasite needs to find host plants within a few weeks after germination because it lacks functional roots and needs to access water and nutrients from host plants. *Cassytha*, in contrast, can survive for over one month by doing photosynthesis prior to parasitization: its roots can suck water and nutrients from the soil and produce energy by photosynthesis. 

*Cassytha* tends to grow mainly on beaches in tropical and subtropical regions, whereas *Cuscuta* ranges across a wider temperate climate zone (not only beaches) [7]. In the field, *Cassytha* can even parasitize trees with highly lignified stems. In contrast, the hosts of *Cuscuta* are typically herbaceous plants with softer stems. Only a few studies have compared the two with regard to the plant interactions. Several plant interaction studies are available on *Cuscuta* [8,9,10], but virtually none on *Cassytha* plant interactions. This calls for dedicated research with a comparison of these parasitic plant interactions. 

For comparison, we chose interactions that are characterized by rapid growth of the parasite. Rapid parasite growth implies successful parasitization of the host plant. This also supports the sampling for chemical analyses and simplifies the observation of morphological changes. For this reason, we focus on the interaction between *Cuscuta japonica* and *Momordica charantia* and on that between *Cassytha filiformis* and *Ipomoea pes-caprae*. In a previous study, we reported that *Cuscuta japonica* parasitizing *Momordica charantia* can grow rapidly, causing hypertrophy and vascular induction at the *Momordica* stem [11]. *Ipomoea pes-caprae* is widely distributed along subtropical coastlines and is one of the most common host plants for *Cassytha filiformis* [12]. Considering the wide and rapid distribution of *Cassytha* at subtropical beaches, *Ipomoea* is an optimal host plant for this study. In addition, we compare hyperparasitization, i.e., *Cuscuta* parasitizing *Cassytha*, based on reports that *Cuscuta* can parasitize *Cassytha* in the field [13]. Metabolite profiling in this highly interesting interaction should help us to understand how parasitic plants themselves respond to other parasites. 

Our investigation is based on metabolite analyses because metabolites and small peptides reportedly translocate between parasitic and host plants [14]. Genome data of these plant species are still limited and no whole genome sequence data are available. Metabolite analyses are therefore the most realistic approach to understanding these interactions. Based on a report that macromolecules (e.g., mRNA and peptides) can translocate between *Cuscuta* and host plants [15], certain metabolites or macromolecules in the parasite could translocate into the host, triggering hypertrophy and subsequent vascular induction. 

Investigating metabolites calls for focusing on certain targets. Firstly, nutrient absorption and effective energy production (i.e., ATP (adenosine triphosphate) production) [16] are key issues in understanding parasitic plant interactions and rapid parasite growth. One approach is to compare the energy charge rate in both species with and without parasitization. Thus, the photosynthetic ability and presence of roots in *Cassytha* might be reflected in the energy production level. Polar metabolites are generally water-soluble substances, which cover saccharides, polyols, amino acids and organic acids. Saccharides and polyols are used as energy and also play a role in stress responses. Saccharides are a key energy source that can be directly translocated from host to parasite [8]. Polyols are known to control osmotic balance (e.g., sorbitol and pinitol), and the polyol quantity can change during plant interactions [17,18]. Some organic acids (e.g., malate) are related to energy metabolism (e.g., TCA (tricarboxylic acid cycle)) and their quantity can be influenced by plant interactions. 

In addition to these target metabolites, we analyzed steroids. Steroids can be upstream of some plant hormones (i.e., brassinolide) and also play a role in the immune system [19,20]. As plant interactions provoke hormonal changes and pathogenic responses, it is interesting to conduct steroid profiling. In previous experiments, we found that *Cuscuta* possesses steroids in high abundance. Some studies have investigated steroids in *Cassytha*, but no comparisons have been made in the past. Methodologically, we used UFLC coupled with a PAD (photo diode array detector) for energy metabolites (i.e., AMP (adenosine monophosphate), ADP (adenosine diphosphate), ATP), and GC-MS for polar metabolite (e.g., sugar and polyols) and steroid analyses. 

## 2. Results

### 2.1. Energy Metabolite Analysis

**Figure 1:**
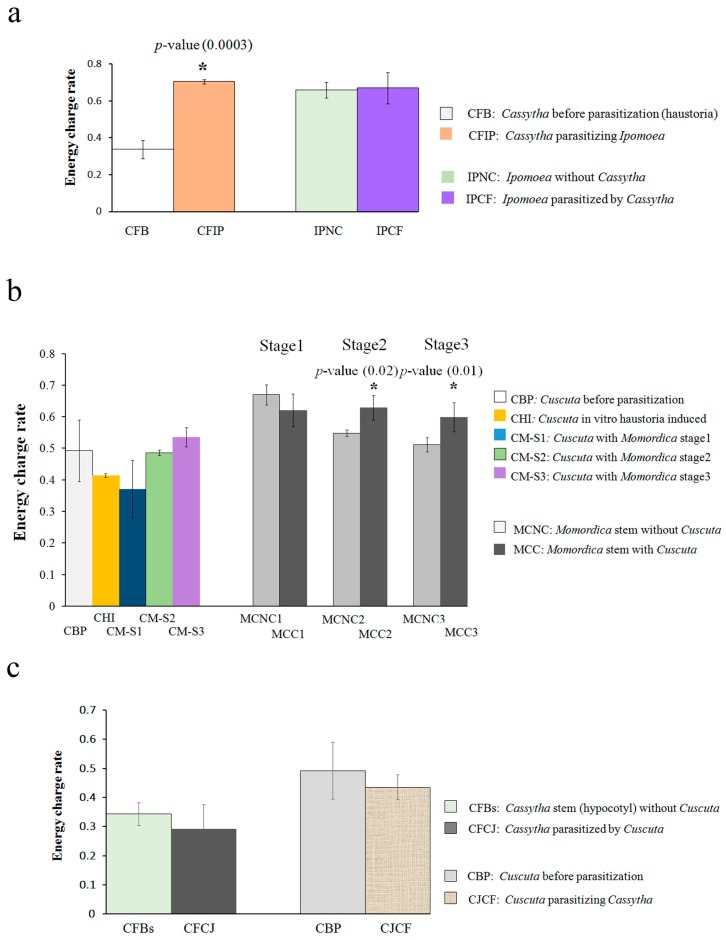
Energy charge of *Cassytha–Ipomoea*, *Cuscuta–Momordica* and *Cassytha–Cuscuta* interaction: energy charge rate data were obtained with UFLC. Value at y axis indicates energy charge rate = ($\frac{\left[\mathrm{ATP}\right]+0.5X\left[\mathrm{ADP}\right]}{\left[\mathrm{ATP}\right]+\left[\mathrm{ADP}\right]+\left[\mathrm{AMP}\right]}$) (*n* = 3 as biological replicates). (**a**) *Cassytha* (haustoria forming part)-*Ipomoea* (stem as parasitized part) interaction; (**b**) *Cuscuta* (haustoria-forming part)-*Momordica* (stem as parasitized part) interaction; (**c**) *Cuscuta* (haustoria-forming part)-*Cassytha* (hypocotyl as parasitized part) interaction; CFB, *Cassytha* before parasitization; CFIP, *Cassytha* parasitizing *Ipomoea*; IPNC, *Ipomoea* without *Cassytha*; IPCF, *Ipomoea* parasitized by *Cassytha*; CBP, *Cuscuta* seedlings before parasitization; CHI, in vitro haustoria-induced; CM-S1, parasitizing *Momordica* at stage 1; CM-S2, parasitizing *Momordica* at stage 2; CM-S3, parasitizing *Momordica* at stage 3; MCC1-3, *Momordica* epicotyls with *Cuscuta* from stage 1 to 3; MCNC1-3, without *Cuscuta* from stage 1 to 3; CFBs, *Cassytha* stem (hypocotyl) without *Cuscuta*; CFCJ, *Cassytha* parasitized by *Cuscuta*; CJCF, *Cuscuta* parasitizing *Cassytha*. * indicates statistical significance compared with negative control. *p*-Value (*t*-test) showed statistical significance between samples. Error bar indicates standard deviation (SD).

The energy charge of *Cassytha* seedlings before parasitization was extremely low (0.33 (±0.05)), but drastically increased after parasitizing *Ipomoea* (0.71 (±0.01)) (*p*-value 0.0003) (Figure 1a). An increase in the ATP/AMP and ADP/AMP ratios contributed to this increase. 

The energy charge value of *Ipomoea* was not changed by *Cassytha* parasitization. The value of *Ipomoea* with and without *Cassytha* parasitization was 0.67 (±0.08) and 0.66 (±0.04), respectively. Both the ATP/AMP and ADP/AMP ratios were higher than 1 both with and without parasitization.

In the case of *Cuscuta*-*Momordica* parasitization, the energy charge of *Cuscuta* before parasitization was 0.49 (±0.09) and there was no statistically significant change after parasitization (Figure 1b). In fact, no *Cuscuta* sample reached a value over 0.6. The value of the *Momordica* stem was over 0.6 at stage 1 (*Momordica* without *Cuscuta* was 0.67 (±0.03) and that with *Cuscuta* was 0.62 (±0.05)). The value of the negative control (without parasitization) fell over time, while that with *Cuscuta* parasitization retained the same constant value (*p*-values of stage 2 and 3 were 0.02 and 0.01, respectively). The ratios ATP/AMP and ADP/AMP were higher than 1. In the negative control, both ratios decreased after stage 2. In contrast, the respective ratios in *Momordica* with parasitization retained the higher value. 

We also investigated the energy charge of hyperparasitization, i.e., *Cuscuta* parasitization of *Cassytha*. The values for both *Cassytha* and *Cuscuta* did not differ from the negative control (before parasitization) (Figure 1c). The energy production rate of *Cuscuta* was not influenced, just as observed in the *Cuscuta–Momordica* interaction. 

### 2.2. Steroid Profiling

Steroid analysis by GC-MS was performed on four plant species (*Cassytha*, *Ipomoea*, *Cuscuta*, *Momordica*). Here, we conducted a relative quantification of selected steroids: campesterol, stigmasterol, *β*-Sitosterol, *β*-Amyrin, lanosterol, and the peak annotated as cycloartenol. 

In the *Cassytha* and *Ipomoea pes–caprae* interaction, stigmasterol and *β*-Sitosterol yielded abundant peaks in the GC chromatogram. The peak annotated as cycloartenol was detected only on *Ipomoea*. In contrast, lanosterol and *β*-Amyrin were undetectable in both species. Although the profiled steroid pattern was not influenced in either *Cassytha* or *Ipomoea*, the absolute abundance of these steroids tended to fall after parasitization in both genera (Supplementary Materials Figure S1a,b). 

**Figure 2:**
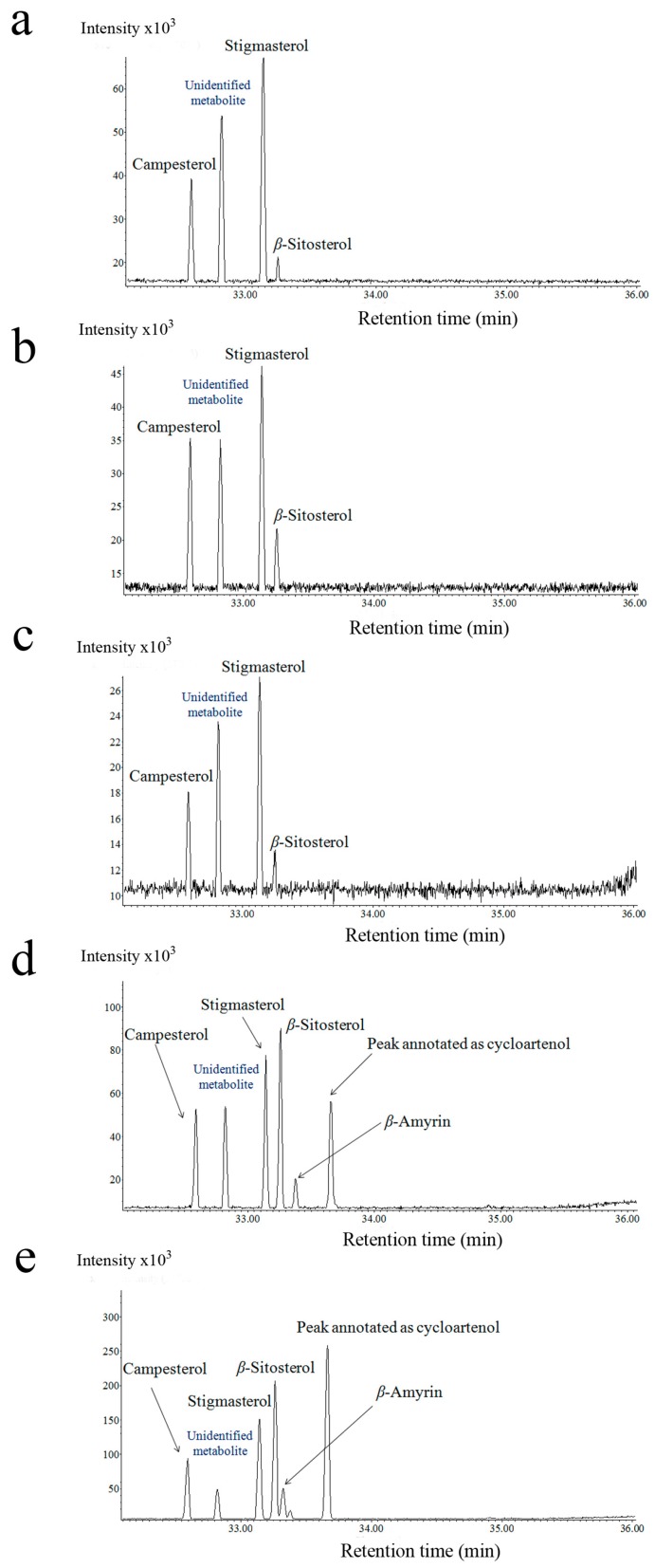
GC chromatogram of steroid profiling of *Cuscuta* haustoria-forming part. (**a**) *Cuscuta* seedlings before parasitization; (**b**) haustoria induced in vitro; (**c**) parasitizing *Momordica* at stage 1; (**d**) parasitizing *Momordica* at stage 2; (**e**) parasitizing *Momordica* at stage 3. Campesterol, stigmasterol, sitosterol, *β*-Amyrin and peak annotated as cycloartenol. In particular, *β*-Amyrin and the peak annotated as cycloartenol increased in intensity after interaction with *Momordica*. Values at *x* and *y* axis indicate retention time and relative intensity, respectively.

With regard to the *Cuscuta–Momordica* interaction, the steroids analyzed by GC-MS were at very low concentrations, and some of them (e.g., cycloartenol) were not detectable in *Momordica*. In contrast, the profiling data of *Cuscuta* showed that steroids made up a relatively big proportion compared with the other lipids (e.g., fatty acid methyl esters (FAMEs)) profiled by GC-MS, compared with those in *Momordica*. Campesterol, stigmasterol and *β*-Sitosterol were detected in all *Cuscuta* samples (Figure 2). In addition, we also detected peaks annotated as cycloartenol in stage 2 and 3, and lanosterol in stage 3. Both cycloartenol and lanosterol were not detectable in *Cuscuta* seedlings before parasitization or in those haustoria induced in vitro. The annotation of steroids was based on the retention time and fragmentation pattern of EI-MS spectra, as well as on the molecular ions of FI-MS spectra (Supplementary Materials Figure S2).

**Figure 3:**
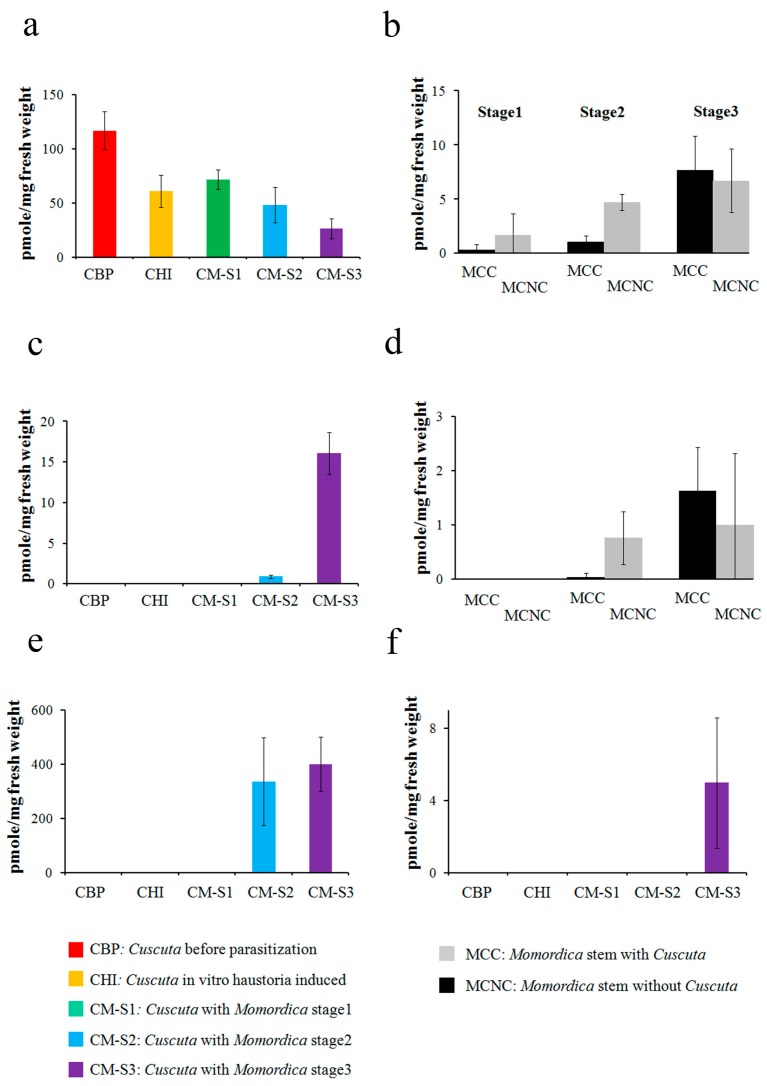
Steroid concentration at *Momordica* stem and *Cuscuta* haustoria-forming part: steroid quantification data were obtained with GC-EI-MS. Value at *y* axis indicates concentration (pmole/gram fresh weight) (*n* = 3 as biological replicates). (**a**) Stigmasterol of *Cuscuta*; (**b**) stigmasterol of *Momordica*; (**c**) *β*-Amyrin of *Cuscuta*; (**d**) *β*-Amyrin of *Momordica*; (**e**) peak annotated as cycloartenol in *Cuscuta*; (**f**) lanosterol of *Cuscuta*. Comparisons were made between *Cuscuta* samples and *Momordica* epicotyls. CBI, *Cuscuta* seedlings before parasitization; CHI, in vitro haustoria-induced; CM-S1, parasitizing *Momordica* at stage 1; CM-S2, parasitizing *Momordica* at stage 2; CM-S3, parasitizing *Momordica* at stage 3; MCC1-3, *Momordica* epicotyls with *Cuscuta* from stage 1 to 3; MCNC1-3, without *Cuscuta* from stage 1 to 3. Error bar indicates standard deviation (SD).

As *Cuscuta* steroid profiling changed conspicuously with parasitization, we also conducted an absolute quantification of the *Cuscuta–Momordica* interaction (Figure 3). 

*Cuscuta* parasitizing *Momordica* showed that the peaks annotated as cycloartenol and *β*-Amyrin started increasing in intensity after stage 2. At stage 2, *Cuscuta* possessed 336.2 (±162.3) cycloartenol and 0.89 (±0.22) (pmole/mg fresh weight) *β*-Amyrin. The values reached 400.4 (±100.8) and 16.1 (±2.6), respectively, at stage 3. At stage 3, 4.9 (±3.6) (pmole/mg fresh weight) lanosterol was detected. In contrast, campesterol and stigmasterol decreased with haustoria induction and a further decrease was recorded with parasitization on *Momordica*. In particular, stigmasterol dropped from 116.6 (±17.7) (before parasitization) to 26.3 (±9.4) at stage 3 (pmole/mg fresh weight) (*p*-value of *t*-test 0.001). In *Momordica*, both *β*-Amyrin and stigmasterol showed lower concentrations and also showed no trends over time or with parasitization (Figure 3). 

In hyperparasitization, campesterol, stigmasterol and *β*-Sitosterol were the steroids detected in *Cassytha*. These were increased by *Cuscuta* parasitization (Supplementary Materials Figure S1c). In the case of *Cuscuta* parasitizing *Cassytha*, the peak annotated as cycloartenol was observed on *Cuscuta* after parasitization (Supplementary Materials Figure S1d). In contrast, campesterol and *β*-Amyrin were low in intensity and lanosterol was undetectable.

### 2.3. Saccharide (Polar Metabolite) Analysis by GC-MS

In the *Cassytha* and *Ipomoea* interaction, sugars and polyols were abundant metabolites in our analysis. In *Cassytha* samples, galactitol was one of the most characteristic and abundant metabolites. Laminaribiose was not detectable in these samples. The absolute abundance of most of *Cassytha*’s polar metabolites tended to drop after parasitization (e.g., malate, fructose, glucose, galactitol, *Myo*-Inositol, sucrose) (Supplementary Materials Figure S3a). For example, fructose, glucose, sucrose and galactitol dropped from 42.6 (±14.7) to 3.2 (±1.5), 41.4 (±14.3) to 2.7 (±1.2), 7.4 (±1.4) to 1.2 (±0.5), 123.9 (±40.5) to 9.3 (±3.2) (nmole/mg fresh weight), respectively (*p*-values of *t*-test 0.01, 0.01, 0.0002, 0.01, respectively).

The absolute abundance of *Ipomoea* polar metabolites—sugars that serve as an energy source—was also influenced by *Cassytha* parasitization. Indeed, the absolute amount of fructose, glucose, and sucrose dropped after *Cassytha* parasitization (Supplementary Materials Figure S3b). In contrast, only galactitol in the *Ipomoea* stem increased after parasitization (*p*-value 0.03), from 0.05 (±0.03) to 0.94 (±0.05) (nmole/mg fresh weight). Other metabolites such as pinitol, quinate and organic acids (e.g., malate and citrate) did not change significantly. 

**Figure 4:**
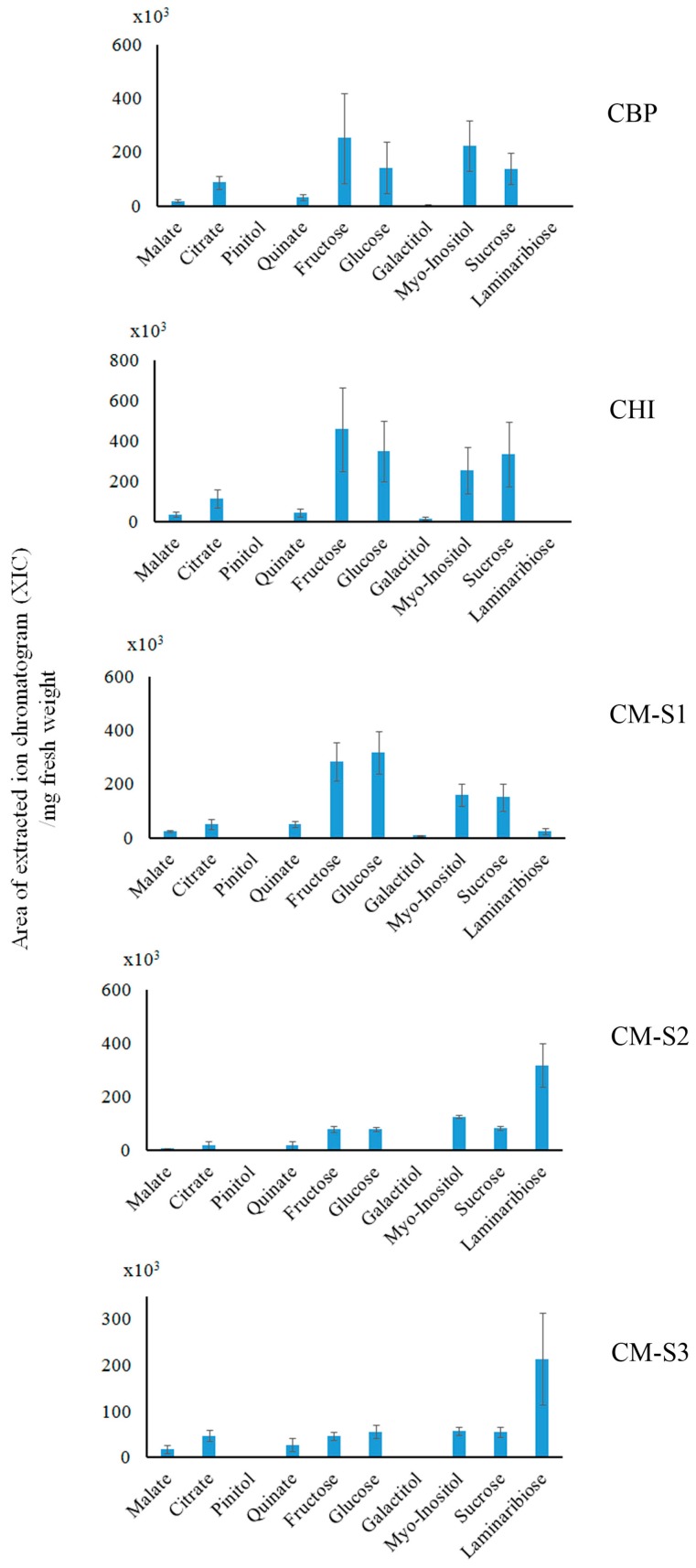
Relative quantification of polar metabolites at *Cuscuta* haustoria-forming part: polar metabolites quantification data were obtained with GC-EI-MS. Value at y axis indicates peak area of extracted ion chromatogram (XIC)/mg fresh weight (*n* = 3 as biological replicates). CBP, *Cuscuta* seedlings before parasitization; CHI, in vitro haustoria-induced; CM-S1, parasitizing *Momordica* at stage 1; CM-S2, parasitizing *Momordica* at stage 2; CM-S3, parasitizing *Momordica* at stage 3. Increase of lamininaribiose at both stage 2 and 3 showed statistical significance. *p*-Value (*t*-test) of laminaribiose in CBP (or CHI) vs. CM-S2 and CBP (or CHI) vs. CM-S3 was 0.004 and 0.02, respectively. Error bar indicates standard deviation (SD).

With regard to the *Cuscuta–Momordica* interaction, only the laminaribiose increase in *Cuscuta* was outstanding and strongly correlated with parasitization to *Momordica* (Figure 4). It increased from 0.01 (±0.01) (nmole/mg fresh weight) (both *Cuscuta* before parasitization as well as in vitro haustoria-induced seedling) to 1.24 (±0.31) (nmole/mg fresh weight) at stage 2 and 0.83 (±0.38) (nmole/mg fresh weight) at stage 3. The annotation of laminaribiose was based on the retention time and fragmentation pattern of the EI-MS spectra, as well as those of the FI-MS spectra (Supplementary Materials Figure S4). In *Momordica*, there was no clear trend in the influence of *Cuscuta* parasitization over time.

In hyperparasitization, malate, pinitol and quinate in *Cassytha* increased after *Cuscuta* parasitization (Supplementary Materials Figure S3c) (*p*-value 0.007, 0.01, 0.02, respectively). For example, pinitol and quinate increased from 0.004 (±0.001) to 0.01 (±0.002) (nmole/mg fresh weight) and from 0.001 (±0.0009) to 0.1 (±0.03) (nmole/mg fresh weight), respectively. Nonetheless, the changes in sugar and polyols were not statistically significant. In *Cuscuta*, no metabolite changed significantly after parasitization. The absolute amount of laminaribiose remained unchanged by parasitization. 

## 3. Discussion

### 3.1. Cassytha–Ipomoea Interaction

The absolute amounts of most polar metabolites and steroids in *Cassytha* decreased after parasitization. A plausible explanation is that water taken from the host plant and a possible lignification process mainly contributed to an increase in fresh weight. As a result, the absolute amount of many metabolites appeared to drop. At the same time, the energy charge of *Cassytha* initiated from a low level, but the value drastically increased after parasitization. Such an enhanced energy production rate would contribute to further elongation and development of *Cassytha* seedlings. 

The data on the polar metabolites suggested that metabolites in *Ipomoea* are partly sucked up by *Cassytha*, as the absolute amount of sugars (fructose, glucose, sucrose) and steroids decreased after parasitization. The smaller energy charge changes in *Ipomoea* after parasitization indicate that *Ipomoea* was relatively tolerant to losing metabolites and water to *Cassytha*. Another point is that the pinitol and quinate amounts in *Ipomoea* were not influenced by parasitization. This indicates that water loss by parasitization did not lead to an increase in pinitol or in any other polyol (e.g., inositol). Parasitization did not cause severe pathogenic responses such as HR (hypersensitive response) or SAR (systemic acquired resistance) in *Ipomoea*. No significant change in quinate also fits this result. Concomitantly, *Ipomoea* parasitized by *Cassytha* in the field does not appear to be lethal, although the growth and flowering rate of *Ipomoea* was negatively influenced. 

### 3.2. Cuscuta–Momordica Interaction

We detected steroids in *Cuscuta* samples but rarely in *Momordica* stems. *Cuscuta* changed its internal steroid proportion and amounts during haustoria formation and during the interaction with *Momordica*. Stigmasterol levels, for example, dropped during haustoria formation. A compound annotated as cycloartenol increased three days after parasitization. As *Momordica* steroids were mostly undetectable, the steroid increase in *Cuscuta* is probably synthesized by *Cuscuta* itself after parasitization. 

In *Cuscuta*, the laminaribiose increase was conspicuous. As laminaribiose was not abundant in *Momordica*, it does not appear to be due to translocation from *Momordica*. A role of laminaribiose in *Cuscuta* was uncertain, but it might be utilized as an osmoprotectant as in other plants [21]. Despite the rapid growth after parasitization to *Momordica*, the energy charge of *Cuscuta* was not altered. One plausible explanation is that an actual increase of ATP production was masked by rapid utilization of ATP in *Cuscuta*. 

In *Momordica*, most of the polar metabolites and steroids did not show a consistent change with parasitization over time. As in the *Cassytha–Ipomoea* interaction, pinitol and quinate were not influenced by parasitization. Indeed, neither HR nor SAR were observed in this interaction [11]. Nonetheless, the energy charge differed. Interestingly, *Momordica* stems without parasitization showed a decreased energy charge rate, but those parasitized by *Cuscuta* maintained the same level. One interpretation is that a development including lignification occurred in the *Momordica* stem, and the overall cell activity at the stem would have dropped accordingly. At the same time, hypertrophy and new vascular formation accompanied cell elongation and proliferation. For this reason, parasitized *Momordica* would need to maintain a higher energy rate. 

### 3.3. Cuscuta–Cassytha Interaction

Hyperparasitization did not alter the energy production rate change in either partner. In *Cuscuta*, the change in energy charge did not depend on the host plant. *Cassytha* as a host plant, in turn, could not enhance its energy production rate. Most likely, the translocation of water and nutrients is exclusively from *Cassytha* to *Cuscuta*. 

The pinitol increase in *Cuscuta* parasitization implies that *Cassytha* was under drought stress and suffered water loss. The quinate increase can be interpreted as a potential pathogenic response to *Cuscuta*. 

### 3.4. Plant Interaction Comparison

The plant interaction differs considerably between *Cuscuta* and *Cassytha*. *Cassytha* possesses roots and can survive by carrying out photosynthesis over one month without parasitization, whereas the photosynthesis ability of *Cuscuta* is quite low and it needs to find a host plant within one week to obtain water and nutrients. The differences in metabolite profiling between the *Cassytha* and *Cuscuta* plant interaction probably reflect such lifestyle differences. Because of its highly reduced scale leaves and reduced roots, energy production is ineffective in *Cassytha*. Consequently, it would need to reduce energy consumption before parasitization and survive by photosynthesis the same as an autotrophic plant [22]. After parasitization, *Cassytha* can obtain nutrients from host plants and can utilize energy just as other non-parasitic plants. This is consistent with a fact that the energy charge rate increased after parasitization. *Cuscuta* seedlings, in contrast, lack roots and cannot survive as autotrophic plants. They require immediate parasitization and rapid growth. A lower energy charge would mean rapid energy consumption. The constant energy charge level in *Cuscuta* could be due to high energy consumption even after parasitization. In fact, higher energy consumption than other autotrophic plants also agrees with rapid growth after parasitization. Apparently, the presence or absence of roots influences such an energy consumption strategy. 

Steroids are another type of metabolite that reflect the difference between *Cassytha* and *Cuscuta*. *Cuscuta* steroids changed proportionally after parasitization, whereby the increase of cycloartenol was an especially characteristic change. The steroid cycloartenol is present in small amounts in many plants [23]. The cycloartenol pathway is known to be present in parasitic plants (i.e., *Cuscuta* and *Orobanche*), and large amounts of cycloartenol and its derivatives are present in *Cuscuta* [19,24]. Those studies analyzed only the parasitic plant attachment to host plants, but did not compare parasitic plants before and after parasitization. We confirmed the presence of cycloartenol and an increase in its derivatives after parasitization to the host plant. 

The peak annotated as cycloartenol also appeared in *Cuscuta* parasitizing other host plants such as *Pisum sativum* [25]. Furthermore, the same peak was also observed in *Ipomoea*, implying a phylogenetic relationship within the family Covolvulaceae, which includes the genus *Cuscuta*. In contrast, in *Cassytha* parasitizing *Momordica*, cycloartenol did not increase in *Cassytha* during the interaction (unpublished data). In that interaction, *Cassytha* grew very slowly, and no hypertrophy or new vascular formation was observed in *Momordica*. There are some reports on steroids in *Cassytha* leaves (steroids in saponin form) [26], but steroids do not appear to be important for their parasitic strategy. 

Considering that the steroid pattern commonly changed in parasitizing *Cuscuta*, the function of cycloartenol and other steroids does not appear to be specifically related to hypertrophy or vascular formation in *Momordica*. As cycloartenol is known to be upstream in the brassinolide synthetic pathway, we tested the effect of brassinolide and cycloartenol on vascular induction in *Momordica* stems: there was no significant influence (unpublished data). 

Steroids (e.g., brassinolides) reportedly have inhibitory effects on plant immune systems [20,27]. Thus, the proportional steroid change in *Cuscuta* haustoria after parasitization might rather be related to inhibition of pathogenic responses by host plants (i.e., anesthetization). Indeed, *Cuscuta* does successfully parasitize many different host species without causing serious defensive responses (e.g., HR and SAR). Another possibility is the utilization of steroids for vascular formation in *Cuscuta* itself. During haustoria development in host plants, the vascular system is newly developed between the *Cuscuta* stem and pore at the apex of haustoria for nutrient transport from the host to *Cuscuta* [28]. The proportional change of *Cuscuta* steroids might influence this. 

Pinitol and quinate (and their ester derivatives) are correlated with drought stress and potential pathogenic responses, respectively [29,30,31]. Thus, we previously found that pinitol increased in Fabaceae after *Cuscuta* parasitization [32]. No pinitol increase in host plants was observed in *Ipomoea* and *Momordica* (only in the host *Cassytha*). Pinitol is a drought stress indicator in *Cassytha* and its increase in hyperparasitization reflects water loss in *Cassytha*. Quinate changed neither in the *Ipomoea* or *Momordica* host, but did increase in *Cassytha* as a host plant. This suggests that *Cuscuta* is recognized as a pathogen by *Cassytha*. Nonetheless, no HR or SAR was found. Accordingly, *Cuscuta* could avoid a serious defensive response by *Cassytha*, much like by many other host plants. Interestingly, *Cassytha* as a host could not completely defend itself against the other parasitic plant, although *Cassytha* as a parasite would also have some system to avoid defensive responses by their host plants.

With regard to saccharides and polyols, there was no significant change correlated with the energy charge change. Nonetheless, galactitol in *Cassytha* was a characteristic, abundant metabolite: galactitol (dulcitol) is typically found in Celestraceae [33] and also in *Cassytha* [34]. The actual role and function of galactitol in these higher plants, however, are poorly understood beyond the correlation with boron transport and polyol synthesis [35]. Based on studies on polyol transport in parasitic plants [17,18], galactitol might also have been used to control the osmotic pressure in the parasitic plant during the interaction. 

In conclusion, stem parasitic plants in the present study showed different metabolite profiling and changes during parasitization. Such differences no doubt reflect their different lifestyles and their morphological differences. 

## 4. Materials and Methods

### 4.1. Plant Culture and Sample Preparation

*Cuscuta japonica* seeds were germinated with concentrated sulfuric acid [11]. Seven days after germination (about 10 cm length), the seedlings were used for parasitization. The in vitro haustoria induction method was used according to previous descriptions [11]. *Momordica charantia* seeds (purchased from Yae Nougei Company, Nagasaki, Japan in 2015) were germinated in flower pots filled with vermiculites. Plants were incubated in a greenhouse with daily watering; the temperature was kept at 30 °C and light followed a 12 h light and dark cycle. The 7-day-old *Cuscuta* seedlings were attached with surgical tape to12-day-old *Momordica* seedling epicotyl stems. Sampling was done as previously described [11]. *Cuscuta* haustoria attaching to *Momordica* stems (stage 1–3: stage 1 (30–36 h after attachment), stage 2 (5 days after attachment), and stage 3 (8 days after attachment)) and the *Momordica* epicotyl stems (both with and without *Cuscuta* parasitization) were excised carefully and collected separately (approx. 20 to 100 mg fresh weight). We also used the haustoria-forming region of *Cuscuta* seedlings before parasitization; *Cuscuta* haustoria induced in vitro were used as control. The haustoria-forming region of 7-day-old *Cuscuta* seedlings (before parasitization) is between 3 and 10 mm from the apex, because this region differentiates into haustoria in in vitro haustoria induction experiments (unpublished data). All samples were immediately frozen in liquid nitrogen and then homogenized with quartz beads prior to extraction of metabolites.

*Cassytha* seeds were collected in 2014–2015 at Yaeyama Islands in Japan (24°23′43.86″ N, 123°45′15.3″ E). Seeds were germinated with concentrated sulfuric acid, as for *Cuscuta*, and the outer seed coat then removed. About twenty days after germination (seedlings about 7 cm long) the seedlings were used to represent seedlings before parasitization. *Cassytha filiformis* seedlings parasitizing *Ipomoea pes-caprae* were collected at Yaeyama Islands in Japan (24°23′43.86″ N, 123°45′15.3″ E). *Cassytha* and *Ipomoea* samples were collected approximately two weeks after *Cassytha* coiling on *Ipomoea*. Based on our pilot experiments, we found the haustoria-forming region of 20-day-old *Cassytha* seedlings (before parasitization). It is the epicotyl region and is between 1 and 2 cm from the apex. With regard to the interaction between *Cassytha* and *Ipomoea*, haustoria of *Cassytha* and the stems of *Ipomoea* where haustoria were attached were excised and collected for metabolite analysis. All samples were immediately frozen in liquid nitrogen and then homogenized with quartz beads prior to extraction of metabolites.

### 4.2. Extraction of Energy Metabolites, Polar Metabolites Analysis

Energy metabolites (AMP, ADP, ATP) were extracted with 1 mL 1% TFA solution. As energy metabolites are phosphorylated, enrichment was carried out with 200 µL Titansphere Phos-TiO Tip (5010-21307, GL Science Inc, Tokyo, Japan). Firstly, the TiO_2_ spin column was conditioned with 1 mL 5%NH_3_ and then 1 mL 0.2% TFA solution. The extract was subjected to the spin column and washed with 1 mL 1% TFA. The flow-through fraction was collected for saccharide and polyol analyses. Phosphorylated energy metabolites were eluted with 1 mL 5% NH_3_. Both the flow-through and elution fraction were lyophilized. The lyophilized elution fraction was dissolved into 100 µL ultrafiltered water and loaded to the UFLC. The lyophilized flow-through containing other polar metabolites (saccharides, polyols, organic acids) was derivatized with 10 μL methoxamine-HCl and 40 μL *N*-methyl-*N*-(trimethylsilyl)trifluoroacetamide prior to GC-MS analysis [36].

### 4.3. Extraction of Steroids

We used the lipophilic compound extraction method of Furuhashi and Weckwerth (2013) [37]. Extraction was carried out with 1 mL of MCW solvent (methanol/chloroform/ ultrafiltered water, 5:2:1, *v*/*v*/*v*) with 20 µg C17:0 free fatty acids as internal standard. Methylesterification to the dried apolar (with inter-phase) pellet was done with 500 µL of 0.5 M sodium methoxide in methanol for 90 min at 60 °C (modified from Basconcillo and McCarry, 2008) [38]. The samples were derivatized with *N*-Methyl-*N*-(trimethylsilyl) trifluoroacetamide (MSTFA; Sigma-Aldrich Inc., St. Louis, MO, USA, 394866) prior to GC-MS analysis.

### 4.4. UFLC Condition

For energy metabolite quantification, conventional phosphate buffer with ODS column separation was used (modified from Liu et al., 2006) [39]. UFLC (CBA-20A, SIL-20A; Shimadzu, Kyoto, Japan) was used for analysis. An Inertsil-ODS4 LC column 5µm, 4.6 × 250mm, (5020-03946, GL Science, Japan) was equipped with a pump system (LC-20AD and DGU-20A3; Shimadzu, Kyoto, Japan). Peaks were detected and analyzed at 260 nm by a PDA (photo diode array detector; SPD-20A, Shimadzu, Kyoto, Japan). Mobile phase A consisted of 0.1 M Na_2_HPO_4_ (pH 7.0; adjusted with glacial acetic acid) in ultra-filtered water. Mobile phase B consisted of 10% methanol and 90% of mobile phase A. Air bubbles in the two solutions were removed using an ultrasonic instrument. The elution gradient program was as follows: 0 min 100% A, 0% B; 3 min 100% A, 0% B; 5 min 90% A, 10% B; 7 min 70% A, 30% B, 10 min 50% A, 50% B, 12 min 0% A, 100% B, 18 min 0% A, 100% B, 20 min 100% A, 0% B, 30 min 100% A, 0% B, 35 min 0% A, 100% B, 45 min 0% A, 100% B, 50 min 100% A, 0% B, B, 55 min 100% A, 0% B. The flow rate of the mobile phase was constant at 0.3 mL/min, and oven (CTO-10A, Shimadzu, Japan) temperature was 40 °C. The injection volume was 10 µL. ATP, ADP and AMP in the samples were identified by comparison with authentic standard retention time (ATP at 13.55 min; ADP at 13.9 min; AMP at 14.5 min). Peak height at each retention time was used for quantification. The energy charge was calculated using the following equation.

$$\text{Energy charge}=\frac{\left[\mathrm{ATP}\right]+0.5X\left[\mathrm{ADP}\right]}{\left[\mathrm{ATP}\right]+\left[\mathrm{ADP}\right]+\left[\mathrm{AMP}\right]}$$

### 4.5. GC/MS Condition

For absolute steroid quantification, GC-MS measurements were carried out on a time-of-flight (TOF) mass spectrometer (AccuTOF GCv 4G: JEOL, Akishima, Japan) equipped with 7890A GC (Agilent Technologies, Santa Clara, CA, USA) as previously described [36]). HP-5MS 30 m, 0.30 mm, 0.25 µm (19091S-433, Agilent Technologies, Palo Alto, CA, USA) was used as GC column. The oven temperature gradient for the samples was as follows. After a 4 min, 70 °C isotherm period, the oven was programmed to rise to 340 °C at a rate of 8 °C·min^−1^, then held at 340 °C for 5 min. The temperature of both the GC-MS ion source and transfer line was set at 250 °C. To quantify steroids, we calculated the peak areas (*n* = 3 as biological replicates) of a conventional 70V EI mode extracted ion chromatogram using software (Escrim; JEOL Ltd., Akishima, Japan). We chose the following *m*/*z* due to good linearity in the standard curve: *m*/*z* 129.1 at retention time 32.67 min for campesterol, *m*/*z* 129.1 at retention time 32.9 min for stigmasterol, *m*/*z* 393.4 at retention time 33.2 min for lanosterol, *m*/*z* 129.1 at retention time 33.3 min for sitosterol, *m*/*z* 218.2 at retention time 33.4 min for *β*-Amyrin, and *m*/*z* 135.1 at retention time 33.7 min for the peak annotated as cycloartenol. Normalization was done by C17:0 free fatty acids. 

For steroid relative quantification and polar metabolite analysis, GC-MS measurements were carried out on an Ultra Quad mass spectrometer (JMS-Q1000GC MkII: JEOL Ltd., Akishima, Japan) equipped with 7890A GC (Agilent Technologies). The oven temperature gradient, the temperature of the GC-MS ion source and the transfer line for the samples were the same as for the steroid analysis by GC-ToF MS above. To quantify polar metabolites, we calculated the peak areas (*n* = 3 as biological replicates) of a conventional 70V EI mode extracted ion chromatogram using software (Escrim; JEOL Ltd.). In polar metabolite analysis by GC-MS, relative quantification was done on selected metabolites (malate, citrate, pinitol, quinate, fructose, glucose, galactitol, *Myo*-Inositol, sucrose, laminaribiose) because these were abundant and detected from most of the samples. For analysis, the following *m*/*z* was chosen: *m*/*z* 233 at retention time 14.07 min for malate, *m*/*z* 273 at retention time 18.5 min for citrate, *m*/*z* 217 at retention time 19.07 min for pinitol, *m*/*z* 345 at retention time 19.3 min for quinate, *m*/*z* 103 at retention time 19.42 min for fructose, *m*/*z* 205 at retention time 20.0 min for glucose, *m*/*z* 217 at retention time 20.34 min for galactitol, *m*/*z* 361 at retention time 28.08 min for sucrose, *m*/*z* 204 at retention time 29.05 min for laminaribiose. Normalization was done by C17:0 free fatty acids, which was spiked during derivatization.

## Supplementary Materials

The following are available online at www.mdpi.com/2223-7747/5/4/43/s1.

## Abbreviations

ADP	Adenosine diphosphate
AMP	Adenosine monophosphate
ATP	Adenosine triphosphate
EI	Electron ionization
FAME	Fatty acid methyl ester
FI	Field ionization
GC-MS	Gas chromatography-mass spectrometry
HCl	Hydrochloric acid
HR	Hypersensitive response
ODS	Octadecyl-silica
PAD	Photo diode array detector
SAR	Systemic acquired resistance
TCA	Tricarboxylic acid cycle
TFA	Trifluoro acetic acid
UFLC	Ultra-high performance liquid chromatography

## References

[1] Jørgensen H. (2008). Parasitic Flowering Plants.

[2] Tada Y., Sugai M., Furuhashi K. (1996). Haustoria of *Cuscuta japonica*, a holoparasitic flowering plant, are induced by cooperative effect of far-red light and tactile stimuli. Plant Cell Physiol..

[3] Furuhashi T., Furuhashi K., Weckwerth W. (2011). The Parasitic Mechanism of the Holostemparasitic Plant *Cuscuta*. J. Plant Interact..

[4] Weber J.Z. (1981). A taxonomic revision of *Cassytha* (Lauraceae) in Australia. J. Adel. Bot. Gard..

[5] McNeal J.R., Arumugunathan K., Kuehl J.V., Boore J.L., de Pamphilis C.W. (2007). Systematics and plastid genome evolution of the cryptically photosynthetic parasitic plant genus *Cuscuta* (Convolvulaceae). BMC Biol..

[6] McLuckie J. (1924). Studies in parasitism. A contribution to the physiology of genus *Cassytha*. Proc. Linn. Soc. NSW.

[7] Kokubugata G., Nakamura K., Forster P.I., Wilson G.W., Holland A.E., Hirayama Y., Yokota M. (2012). *Cassytha pubescens* and *C. glabella* (Lauraceae) are not disjunctly distributed between Australia and the Ryukyu Archipelago of Japan—Evidence from morphological and molecular data. Aust. Syst. Bot..

[8] Hibberd J.M., Jeschke W.D. (2001). Solute flux into parasitic plants. J. Exp. Bot..

[9] Cheng X., Ruyter-Spira C., Bouwmeester H. (2013). The interaction between strigolactones and other plant hormones in the regulation of plant development. Front. Plant Sci..

[10] Ranjan A., Ichihashi Y., Farhi M., Zumstein K., Townsley B., David-Schwartz R., Sinha N.R. (2014). De novo assembly and characterization of the transcriptome of the parasitic weed dodder identifies genes associated with plant parasitism. Plant Physiol..

[11] Furuhashi T., Kojima M., Sakakibara H., Fukushima A., Hirai M.Y., Furuhashi K. (2014). Morphological and Plant Hormonal Changes during Parasitization by *Cuscuta japonica* on *Momordica charantia*. J. Plant Interact..

[12] Kokubugata G., Yokota M. (2012). Host Specificity of *Cassytha filiformis* and *C. pergracilis* (Lauraceae) in the Ryukyu Archipelago. Bull. Natl. Mus. Nat. Sci. Ser. B.

[13] Chen M.Y., Tsai J.L., Liao G.I. (1992). The parasitism of *Cassytha filiformis* in Taiwan. Q. J. Chin. For..

[14] Birschwilks M., Sauer N., Scheel D., Neumann S. (2007). *Arabidopsis thaliana* is a susceptible host plant for the holoparasite *Cuscuta* spec.. Planta.

[15] LeBlanc M., Kim G., Westwood J.H. (2012). RNA trafficking in parasitic plant systems. Front. Plant Sci..

[16] Pradet A., Raymond P. (1983). Adenine nucleotide ratios and adenylate energy charge in energy metabolism. Annu. Rev. Plant Physiol..

[17] Richter A., Popp M. (1992). The physiological importance of accumulation of cyclitols in *Visum album* L.. New Phytol..

[18] Wanek W., Richter A. (1993). L-Idiol: NAD 5-oxidoreductase in *Viscum album*: Utilization of host-derived sorbitol. Plant Physiol. Biochem..

[19] Rohmer M., Ourisson G., Benveniste P., Bimpson T. (1975). Sterol biosynthesis in heterotrophic plant parasite. Phytochemistry.

[20] Albrechta C., Boutrot F., Segonzac C., Schwessinger B., Gimenez-Ibanez S., Chinchilla D., Rathjen J.P., de Vries S.C., Zipfel C. (2012). Brassinosteroids inhibit pathogen-associated molecular pattern–triggered immune signaling independent of the receptor kinase BAK1.. Proc. Natl. Acad. Sci. USA.

[21] Vazquez-Duhalt R., Arredondo-Vega B.O. (1991). Haloadaptation of the green alga *Botryococcus braunii* (race a). Phytochemistry.

[22] Abubacker M.N., Prince M., Hariharan Y. (2005). Histochemical and biochemical studies of parasite–host interaction of *Cassytha filiformis* Linn. and *Zizyphus jujuba* Lamk.. Curr. Sci..

[23] Kadkade P.G., Lujan C., Rolz C. (1983). Studies on the distribution of phytosterols in *Dioscorea* species tubers. Z. Naturforsch..

[24] Benveniste P. (1986). Sterol biosynthesis. Annu. Rev. Plant Physiol..

[25] Furuhashi T., Nakamura T., Fragner L., Roustan V., Schon V., Weckwerth W. (2016). Biodiesel and poly-unsaturated fatty acids production from algae and crop plants—A rapid and comprehensive workflow for lipid analysis. Biotechnol. J..

[26] Edewor T.I., Owa S.O., Ologan A.O., Akinfemi F. (2016). Quantitative determination of the saponin content and GC-MS study of the medicinal plant *Cassytha filiformis* (linn.) leaves. J. Coast. Life Med..

[27] Lozano-Duran R., Zipfel C. (2015). Trade-off between growth and immunity: Role of brassinosteroids. Trends Plant Sci..

[28] Hong L., Shen H., Chen H., Li L., Hu X., Xu X., Ye W., Wang Z. (2011). The morphology and anatomy of the haustoria of the holoparasitic angiosperm *Cuscuta campestris*. Pak. J. Bot..

[29] Streeter J.G., Lohnes D.G., Fioritto R.J. (2001). Patterns of pinitol accumulation in soybean plants and relationships to drought tolerance. Plant Cell Environ..

[30] Krasensky J., Jonak C. (2012). Drought, salt, and temperature stress-induced metabolic rearrangements and regulatory networks. J. Exp. Bot..

[31] Murthy P.S., Manonmani H.K. (2009). Physico-chemical, antioxidant and antimicrobial properties of Indian monsooned coffee. Eur. Food Res. Technol..

[32] Furuhashi T., Fragner L., Furuhashi K., Valledor L., Sun X., Weckwerth W. (2012). Metabolite Changes with Induction of *Cuscuta* Haustorium and Translocation from Host Plants. J. Plant Interact..

[33] Loescher W.H. (1987). Physiology and metabolism of sugar alcohols in higher plants. Physiol. Plant..

[34] Plouvier V., Swain T. (1963). The distribution of aliphatic polyols and cyclitols. Chemical Plant Taxonomy.

[35] Noiraud N., Maurousset L., Lemoine R. (2001). Transport of polyols in higher plants. Plant Physiol. Biochem..

[36] Furuhashi T., Ishii K., Tanaka K., Weckwerth W., Nakamura T. (2015). Fragmentation patterns of methyloxime-trimethylsilyl derivatives of constitutive mono- and disaccharide isomers analyzed by gas chromatography/field ionization mass spectrometry. Rapid Commun. Mass Spectrom..

[37] Furuhashi T., Weckwerth W., Weckwerth W., Kahl G. (2013). Introduction to Lipid (FAME) Analysis in Algae Using Gas Chromatography-Mass Spectrometry. Handbook of Plant Metabolomics: Metabolite Profiling and Networking.

[38] Basconcillo L.S., McCarry B.E. (2008). Comparison of three GC/MS methodologies for the analysis of fatty acids in Sinorhizobium meliloti: Development of a micro-scale, one-vial method. J. Chromatogr. B Anal. Technol. Biomed. Life Sci..

[39] Liu H., Jiang Y., Luo Y., Jiang W. (2006). A simple and rapid determination of ATP, ADP and AMP concentrations in pericarp tissue of litchi fruit by high performance liquid chromatography. Food Technol. Biotechnol..

