# Supplementary material for: Analysis of Metabolites in Stem Parasitic Plant Interactions: Interaction of Cuscuta–Momordica versus Cassytha–Ipomoea
Source: Plants (Basel). 2016 Dec 7;5(4):43. doi: 10.3390/plants5040043 (PMC5198103; doi:10.3390/plants5040043)
Supplement: Supplementary file 1 [file plants-05-00043-s001.pdf]

Supplementary Materials: Analysis of Metabolites in Stem Parasitic Plant Interactions: Interaction of *Cuscuta-Momordica* versus *Cassytha-Ipomoea*

Takeshi Furuhashi, Takemichi Nakamura and Koji Iwase

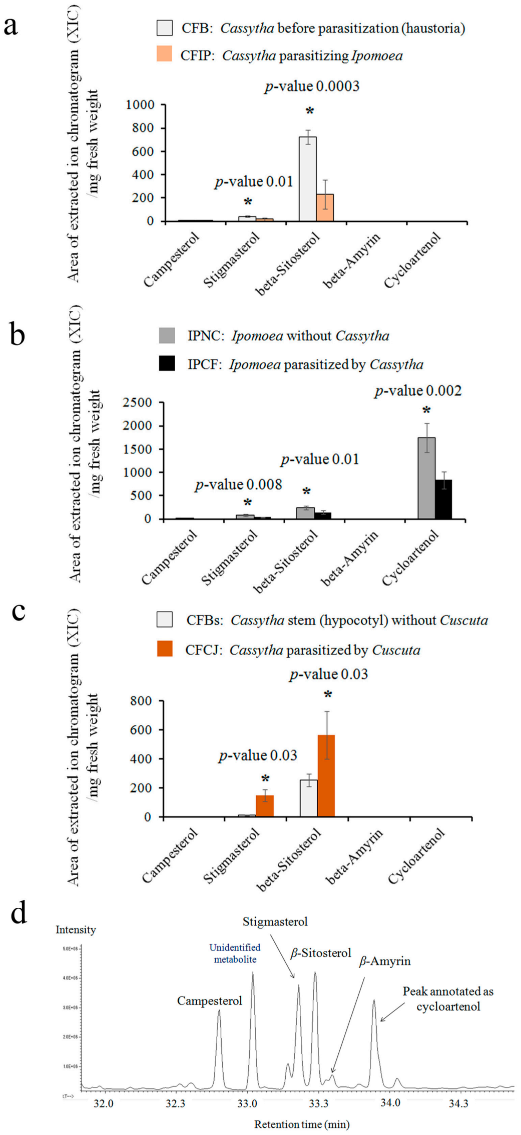

Figure S1. (**a**–**c**) Relative quantification of steroid. (**a**) *Cassytha* (epicotyl as haustoria-forming part) before and after parasitizing *Ipomoea* (stem as parasitized part); (**b**) *Ipomoea* with and without *Cassytha* parasitization; (**c**) *Cassytha* stem (hypocotyl) with and without *Cuscuta* parasitization: steroid quantification data were obtained with GC-EI-MS. Value at y axis indicates peak area of extracted ion chromatogram (XIC)/mg fresh weight (n = 3 as biological replicates). CFB, *Cassytha* before parasitization (haustoria-forming part); CFIP, *Cassytha* parasitizing *Ipomoea*; IPNC, *Ipomoea* without *Cassytha*; IPCF, *Ipomoea* parasitized by *Cassytha*; CFBs, *Cassytha* stem (hypocotyl) without *Cuscuta*; CFCJ, *Cassytha* parasitized by *Cuscuta*. * Indicates statistical significance compared with negative control. p-Value (t-test) showed statistical significance between samples. (**d**) GC chromatogram of steroid profiling of *Cuscuta* haustoria-forming part, parasitizing *Cassytha* stem (hypocotyl). β-Amyrin and a peak annotated as cycloartenol was observed. Values at x and y axis indicate retention time and relative intensity, respectively. Error bar indicates standard deviation (SD).

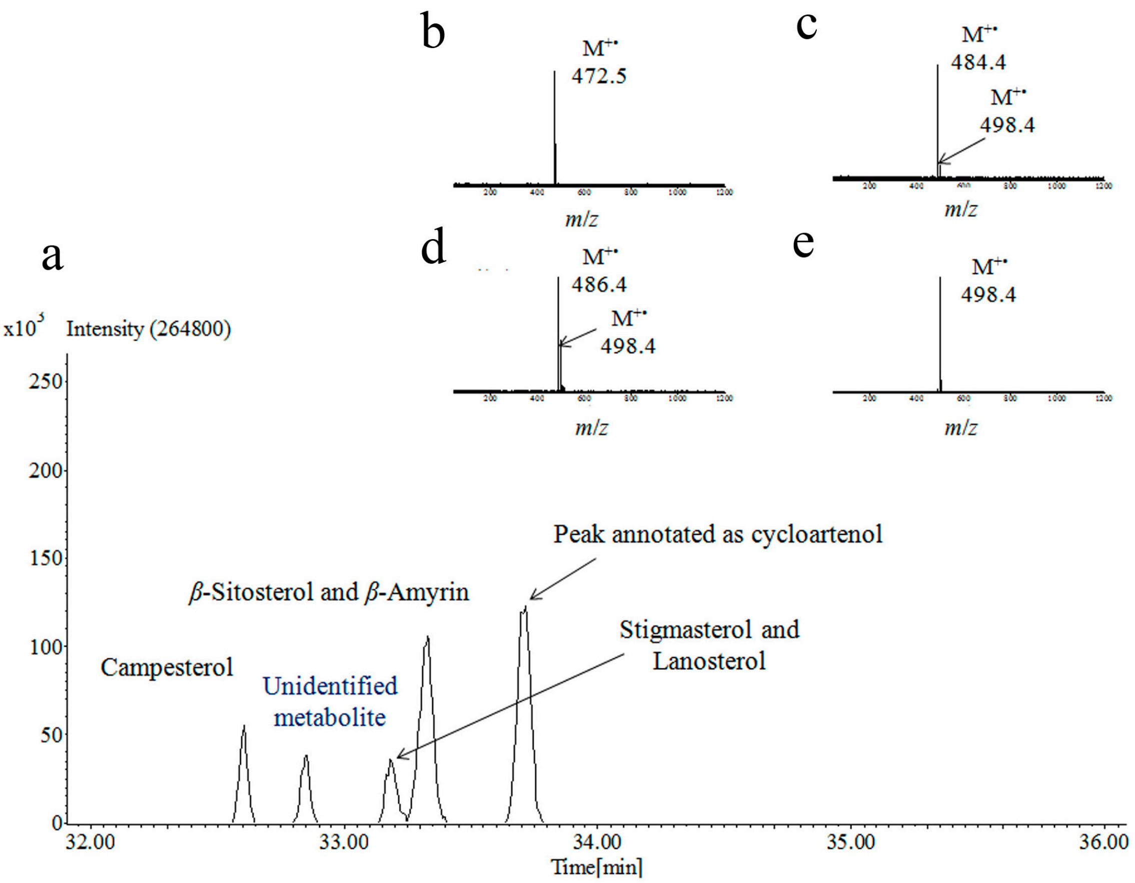

Figure S2. GC chromatogram and MS spectra of *Cuscuta* parasitizing *Momordica* at stage 3. In GC-FI, exclusively molecular ions of steroids (all 1TMS forms) were observed. (**a**) GC-FI-MS chromatogram of *Cuscuta*; (**b**–**e**) MS spectra of each peak; (**b**) campesterol; (**c**) stigmasteol and lanosterol; (**d**) sitosterol and *β*-Amyrin; (**e**) annotated as cycloartenol.

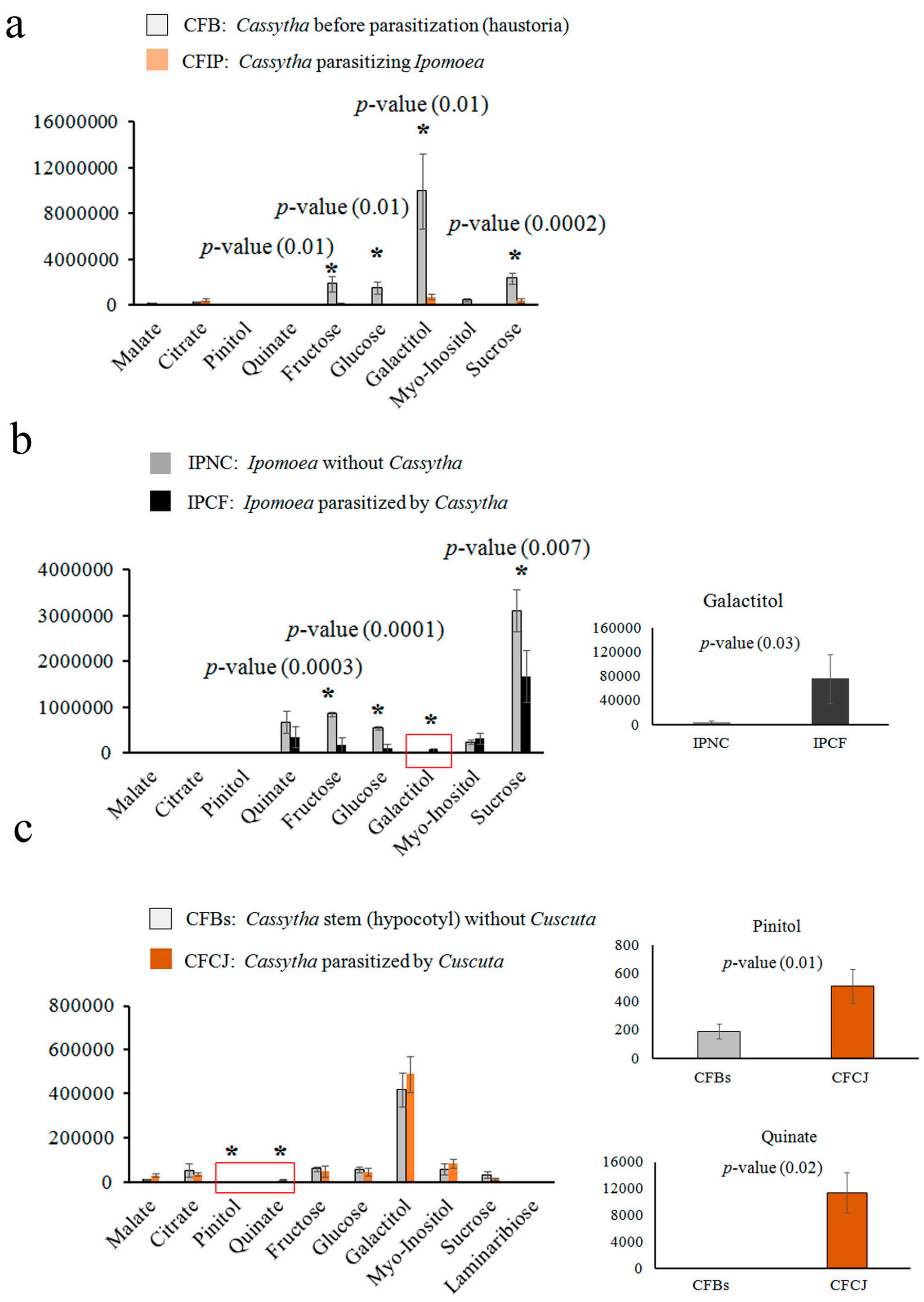

Figure S3. Relative quantification of polar metabolites: (**a**) *Cassytha* (epicotyl as haustoria-forming part) before and after parasitizing *Ipomoea* (stem as parasitized part); (**b**) *Ipomoea* with and without *Cassytha* parasitization; Magnified graph of galactitol is on right side; (**c**) *Cassytha* stem (hypocotyl) with and without *Cuscuta* parasitization. Magnified graph of pinitol and quinate are on right side. Polar metabolites quantification data were obtained with GC-EI-MS. Value at y axis indicates peak area of extracted ion chromatogram (XIC)/mg fresh weight (n = 3 as biological replicates). CFB, *Cassytha* before parasitization (haustoria-forming part); CFIP, *Cassytha* parasitizing *Ipomoea*; IPNC, *Ipomoea* without *Cassytha*; IPCF, *Ipomoea* parasitized by *Cassytha*. CFBs, *Cassytha* stem (hypocotyl) without *Cuscuta*; CFCJ, *Cassytha* parasitized by *Cuscuta*. * Indicates statistical significance compared with negative control. p-Value (t-test) showed statistical significance between samples. Error bar indicates standard deviation (SD).

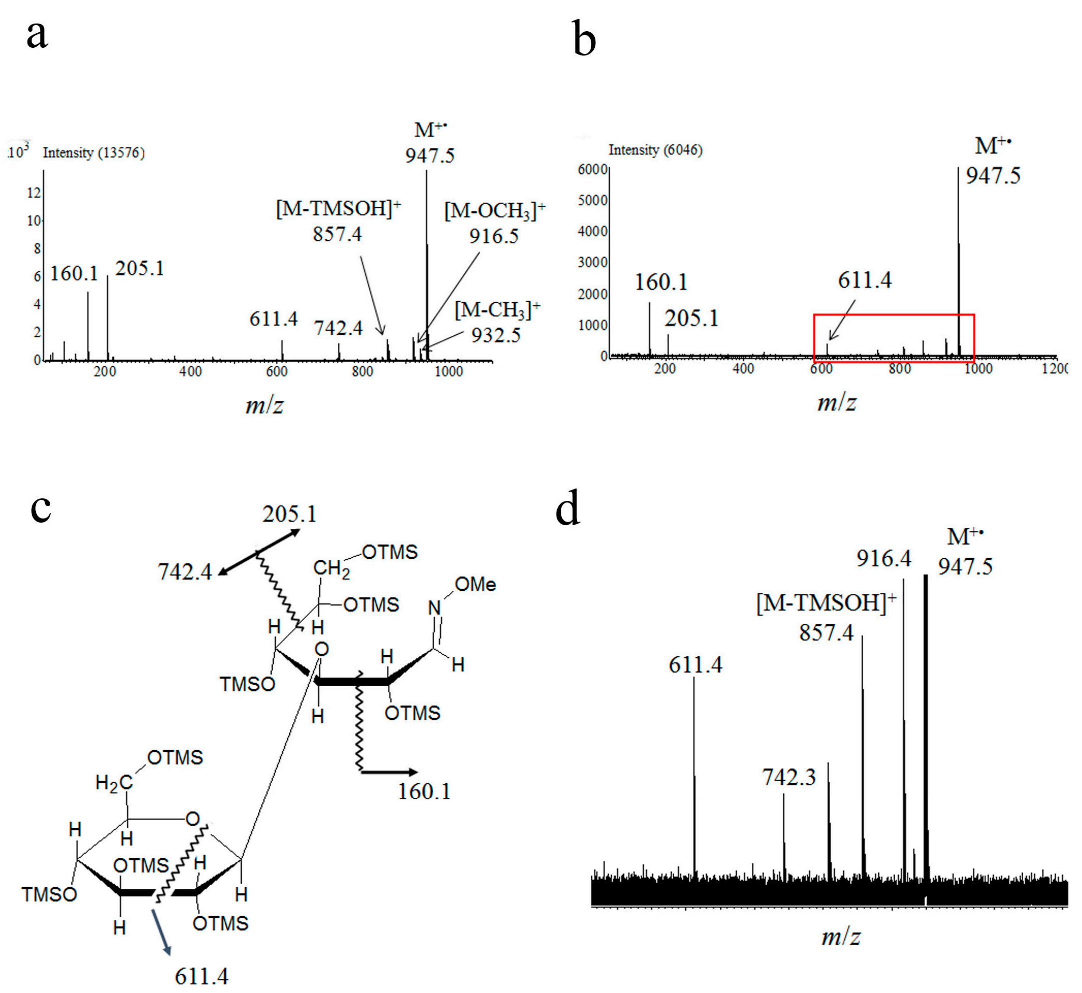

Figure S4. FI-MS spectra of laminaribiose standard and *Cuscuta* parasitizing *Momordica* at stage 3. (**a**) FI-MS spectra of laminaribiose standard; (**b**) FI-MS spectra of peak annotated as laminaribiose in *Cuscuta* sample; (**c**) Fragment assignment of laminaribiose FI-MS spectra; (**d**) Magnified MS spectra of red square of (**b**). In GC-FI-MS, molecular ions of laminaribiose (8TMS forms with 1Methyloxime) as well as characteristic fragment (*m*/*z* 611.4) were observed as *m*/*z* 947.5. No characteristic fragments of other potentially co-eluting isomeric disaccharides (i.e., trehalose, sophorose, maltose), which are *m*/*z* 903.4, 409.2, 787.4, were detected.
